# A Multi-Gene Panel to Identify Lipedema-Predisposing Genetic Variants by a Next-Generation Sequencing Strategy

**DOI:** 10.3390/jpm12020268

**Published:** 2022-02-11

**Authors:** Sandro Michelini, Karen L. Herbst, Vincenza Precone, Elena Manara, Giuseppe Marceddu, Astrit Dautaj, Paolo Enrico Maltese, Stefano Paolacci, Maria Rachele Ceccarini, Tommaso Beccari, Elisa Sorrentino, Barbara Aquilanti, Valeria Velluti, Giuseppina Matera, Lucilla Gagliardi, Giacinto Abele Donato Miggiano, Matteo Bertelli

**Affiliations:** 1 Vascular Diagnostics and Rehabilitation Service, Marino Hospital, ASL Roma 6, 00047 Marino, Italy; sandro.michelini@aslroma6.it; 2 Department of Endocrinology and Research, Total Lipedema Care, Los Angeles, CA 90211, USA; kaherbst@gmail.com; 3 MAGI EUREGIO, 39100 Bolzano, Italy; vincenza_precone@yahoo.it (V.P.); giuseppe.marceddu@assomagi.org (G.M.); elisa.sorrentino@assomagi.org (E.S.); matteo.bertelli@assomagi.org (M.B.); 4 MAGI’S LAB, 38068 Rovereto, Italy; elena.manara@assomagi.org (E.M.); astrit.dautaj@assomagi.org (A.D.); paolo.maltese@assomagi.org (P.E.M.); 5 Department of Pharmaceutical Sciences, University of Perugia, 06123 Perugia, Italy; mariarachele.ceccarini@unipg.it (M.R.C.); tommaso.beccari@unipg.it (T.B.); 6 C.I.B., Consorzio Interuniversitario per le Biotecnologie, 34148 Trieste, Italy; 7 UOC Nutrizione Clinica, Fondazione Policlinico Universitario A. Gemelli IRCCS, 00168 Rome, Italy; barbara.aquilanti@policlinicogemelli.it (B.A.); valeriavelluti@gmail.com (V.V.); giusmatera@yahoo.it (G.M.); lucilla.gagliardi@gmail.com (L.G.); giacintoabele.miggiano@unicatt.it (G.A.D.M.)

**Keywords:** subcutaneaous fat tissue accumulation, lipedema, partial lipodystrophy, NGS

## Abstract

Lipedema is a disabling disease characterized by symmetric enlargement of the lower and/or upper limbs due to deposits of subcutaneous fat, that is easily misdiagnosed. Lipedema can be primary or syndromic, and can be the main feature of phenotypically overlapping disorders. The aim of this study was to design a next-generation sequencing (NGS) panel to help in the diagnosis of lipedema by identifying genes specific for lipedema but also genes for overlapping diseases, and targets for tailored treatments. We developed an NGS gene panel consisting of 305 genes potentially associated with lipedema and putative overlapping diseases relevant to lipedema. The genomes of 162 Italian and American patients with lipedema were sequenced. Twenty-one deleterious variants, according to 3 out of 5 predictors, were detected in *PLIN1*, *LIPE*, *ALDH18A1*, *PPARG*, *GHR*, *INSR*, *RYR1*, *NPC1*, *POMC*, *NR0B2*, *GCKR*, *PPARA* in 17 patients. This extended NGS-based approach has identified a number of gene variants that may be important in the diagnosis of lipedema, that may affect the phenotypic presentation of lipedema or that may cause disorders that could be confused with lipedema. This tool may be important for the diagnosis and treatment of people with pathologic subcutaneous fat tissue accumulation.

## 1. Introduction

Lipedema is characterized by an abnormal and excessive accumulation of subcutaneous fat. It typically affects the lower and/or upper limbs, resulting in a debilitating disease. The extra fat deposition results in tender, painful, and easily bruising tissue. The onset of lipedema is usually at puberty and mostly affects women [1,2,3]. It is known that sex hormones determine the anatomical site of the accumulation of adipose tissue, and this might be related to the higher prevalence of this disease in women than in men [4,5]. The global prevalence of lipedema has been estimated to be around 1–9/100,000 [6]. However, epidemiological data may not be accurate because lipedema can be misdiagnosed and confused with other apparently similar diseases, like obesity and lymphedema. The main difference between lipedema and obesity is that in obesity, fat accumulation is generalized (including on the abdomen early in the disease) with a high body mass index, whereas lipedema is generally limited to the limbs and the body mass index may be normal. However, with respect to lymphedema, lipedema is always bilateral and associated with pain and bruising, while lymphedema can be unilateral and is characterized by a positive Stemmer sign [7,8]. Dieting cannot effectively improve lipedema [8], although it can reduce obesity associated with lipedema. Lipedema can be considered an independent disease or part of a syndrome. Currently, the diagnosis of lipedema is based on medical history, physical examination, blood tests, and imaging techniques [9]. Lipedema can be considered a component of a spectrum of diseases characterized by dysregulated proliferation of adipose tissue and pain, including Dercum disease, with multiple painful nodules on the trunk and limbs that can be accompanied by lipedema [10,11]; and Madelung disease or multiple symmetric lipomatosis, where nodular fat and lipomas occur on the upper part of the body [3,10,11], but may also occur in a distribution similar to lipedema [12].

The genetic etiology of lipedema is not yet fully understood. Genes associated with syndromic subcutaneous fat accumulation have been identified, such as *POU1F1A*, *NSD1*, *ELN*, *FZD9*, *MLXIPL*, and *ALDH18A1* [2]. The molecular causes of isolated lipedema are becoming clearer, and the involvement of the *AKR1C1* gene has been recently reported [13]. Liposuction is the main surgical intervention for lipedema. While conservative therapy may temporarily reduce symptoms, liposuction is able to remove the excess adipose tissue [14]. Potentially, knowing the genetic etiology of lipedema can help in the prediction of the effectiveness of liposuction or other treatments [15]. Currently, no guidelines for the molecular diagnosis of lipedema exist; however, genetic tests may help exclude overlapping conditions. Within this scenario, NGS techniques can improve lipedema diagnosis and management. In fact, NGS can allow for the simultaneous analysis of multiple genes in large cohorts of patients [2,3,16]. The aim of this study was to demonstrate the implementation of an NGS panel for the diagnosis of non-syndromic lipedema, but also evaluate for syndromic forms of lipedema, and to include analysis of candidate genes possibly associated with lipedema, subcutaneous fat tissue accumulation, and fat metabolism that may affect the phenotypic presentation of lipedema.

## 2. Materials and Methods

### 2.1. Study Subjects and Samples

The 162 Italian and US patients recruited for this study received the diagnosis of lipedema according to Wold’s criteria [17,18] and were classified in four stages in accordance with the severity of the phenotype [18]. All patients received genetic counseling to explain the risks and benefits of genetic testing. Patients with lipedema gave written informed consent for participation at the time of recruitment, including the use of their anonymous genetic data for research. The study was performed according to the declaration of Helsinki. Ethical approval and clearance were received from the Ethical Committee of Azienda Sanitaria dell’Alto Adige, Italy (Approval No. 132-2020 of 18 November 2020). A blood EDTA sample was collected from each subject. Samples of genomic DNA of all subjects were extracted from peripheral blood using a commercial kit (SAMAG 120 BLOOD DNA Extraction Kit).

### 2.2. Panel Design

A NGS panel was developed consisting of 305 genes possibly associated with lipedema. The genes included in the panel were associated with lipedema and subcutaneous fat tissue accumulation on the basis of data reported in the Human Gene Mutation Database (HGMD Professional), Online Mendelian Inheritance in Man (OMIM), Orphanet, GeneReviews, and PubMed. The custom DNA probes were designed using Twist Bioscience technology (https://www.twistbioscience.com/, last accessed on 22 December 2021). Illumina NGS sequencing was carried out by an IntegraGen Genomic Service (www.integragen-genomics.com, last accessed on 22 December 2021).

The NGS panel included genomic targets comprising coding exons and 15 bp flanking regions of each gene. The cumulative target length of the gene panel was 839,308 bp. The list of genes possibly associated with lipedema included in the NGS panel is shown in Supplementary Table S1.

### 2.3. Genetic Analysis and Variant Detection

DNA samples were processed using Illumina NovaSeq6000™ (Illumina, San Diego, CA, USA) using a paired-end protocol and reads that were 100 bp long, following the manufacturer’s protocol. Fastq (forward-reverse) files were obtained after sequencing. Bioinformatic analysis was performed as previously described [19]. Briefly, the sequencing reads were mapped to the genome by the Burrow-Wheeler Aligner (BWA version 0.7.17-r1188) software. Duplicates were removed using the SAMBAMBA (version 0.6.7) program and MarkDuplicates GATK tool (version 4.0.0.0). The generated BAM alignment files were refined by local realignment and base quality score recalibration using the RealignerTargetCreator and IndelRealigner GATK tools. We used international databases dbSNP (www.ncbi.nlm.nih.gov/SNP/, last accessed on 22 December 2021), Human Gene Mutation Database professional (HGMD; http://www.biobase international.com/product/hgmd, last accessed on 22 December 2021) and OMIM (https://www.omim.org/, last accessed on 22 December 2021) for the annotation of all nucleotide changes. In silico prediction of the deleteriousness of nucleotide changes in exons was performed using MutationTaster (http://www.mutationtaster.org/, last accessed on 22 December 2021), SIFT (https://sift.bii.a-star.edu.sg/, last accessed on 22 December 2021), Polyphen-2 (http://genetics.bwh.harvard.edu/pph2/, last accessed on 22 December 2021), CADD score (https://cadd.gs.washington.edu/, last accessed on 22 December 2021), and VarSome (https://varsome.com/, last accessed on 22 December 2021). Minor allele frequencies (MAF) were checked in the Genome Aggregation Database (gnomAD-http://gnomad.broadinstitute.org/, last accessed on 22 December 2021). We considered variants that had a MAF < 1% in the general population and were predicted to be deleterious by at least three predicting tools out of five as good candidates. Variants with a CADD score > 15 were considered deleterious.

## 3. Results

In total, 162 patients were analyzed for genes possibly involved in lipedema, subcutaneous fat tissue accumulation, and fat metabolism. Gene panel sequencing generated an evenly distributed depth of coverage >98% at 10X. Twenty-one deleterious variants, according to 3 out 5 predictors, were detected in the *Perilipin 1* (*PLIN1*), *Lipase E* (*LIPE*), *Aldehyde Dehydrogenase 18 Family Member A1* (*ALDH18A1*), *Peroxisome Proliferator-Activated Receptor Gamma* (*PPARG*), *Growth Hormone Receptor* (*GHR*), *Insulin Receptor* (*INSR*), *Ryanodine Receptor 1* (*RYR1*), *Niemann-Pick C Intracellular Cholesterol Transporter 1* (*NPC1*), *Proopiomelanocortin* (*POMC*), *Nuclear Receptor Subfamily 0 Group B Member 2* (*NR0B2*), *Glucokinase Regulator* (*GCKR*) and *Peroxisome Proliferator-Activated Receptor Alpha* (*PPARA*) genes in 17 patients (Table 1).

### 3.1. Variants in Genes Linked to Syndromic Fat Accumulation

In *ALDH18A1*, we identified in two unrelated subjects, two predicted deleterious rare variants, c.2276C>T; p.Thr759Ile (rs781126562) and c.1233G>T; p.Leu411Phe (rs758828421). The amino acid Thr759 is totally conserved among species, whereas Leu411 is not totally conserved but is never substituted with a phenylalanine. *ALDH18A1* encodes an enzyme that catalyzes the reduction of glutamate to deltal-pyrroline-5-carboxylate, an important key point in the de novo biosynthesis of proline, ornithine, and arginine [20]. Germline variants in *ALDH18A1* may cause abnormal fat accumulation in conjunction with cutis laxa type III. Cutis laxa is a group of connective tissue diseases in which skin hangs in loose pendulous folds due to decreased elastic tissue formation. Because lipedema is associated with hypermobile joints, a connective tissue disease, and is found in Williams syndrome [21], *ALDH18A1* should not be ruled out as a gene that influences lipedema.

*GHR* encodes a member of the type I cytokine receptor family, which is a transmembrane receptor for growth hormones [22], and heterozygous mutations in this gene have been associated with partial growth hormone insensitivity and short stature, as in the case of mutations in *POU1F1* that have been linked with growth hormone deficiency and lipedema [23]. Therefore, it is plausible that truncating variants, like the one identified by us, c.293G>A; p.Trp98* (rs1237134960) in *GHR* might be associated with lipedema.

### 3.2. Differential Diagnosis for Lipedema

The variant in *PLIN1*, c.722T>C; p.Leu241Pro (rs914779001) does not have a reported frequency and the amino acid is totally conserved among mammals. Genetic variants in *PLIN1* causes familial partial lipodystrophy (FPLD), type 4. The phenotype of women with FPLD type 4 is different from that of women with lipedema, with upper body obesity compared to gynoid fat deposits, respectively [2]. The presence of this gene mutation might suggest greater scrutiny surrounding the diagnosis of lipedema.

We found the rare variant c.1141C>T; p.Arg381Cys (rs772317492) in *LIPE*. Germline variants in *LIPE* are known to cause a recessive syndrome characterized by multiple symmetric lipomatosis, partial lipodystrophy, and insulin resistance [24]. The presence of this gene variant may also bring into question the diagnosis of lipedema.

The predicted deleterious variant, c.1424C>T; p.Thr475Met (rs1479145908) in *PPARG*, has no reported population frequency [25], but the amino acid is totally conserved among species. Interestingly, this gene, when mutated, causes FPLD type 3, which shows abnormal fat accumulation in the abdomen, and is caused by heterozygous missense mutations in *PPARG* [24,26]. Therefore, this gene can be considered a good candidate to explain the observed phenotype in lipedema.

Heterozygous truncating variants in *POMC* have also been found to be associated with an increased risk of fat accumulation. Therefore, it may be possible that the variant found in our cohort, c.616G>T; p.Glu206* (rs202127120), might be linked with the onset of lipedema [27]. Congenital deficiency of proopiomelanocortin, encoded by *POMC*, results in a syndrome of hypoadrenalism, severe obesity, and altered skin and hair pigmentation. A role for genetic variation in or near the *POMC* locus as a determinant of obesity or obesity-related quantitative traits in the general population has been reported [27,28].

A patient was found to be a carrier of two variants in two good candidate genes: *NR0B2* (c.265C>T; p.Gln89 *-rs150160927) and *GCKR* (c.1135dup; p.Thr379Asnfs * 36-rs573498430). Heterozygous loss-of-function mutations in *NR0B2* in OMIM (*604630) are associated with mild early-onset obesity, whereas truncating mutations in *GCKR* may be linked to increased levels of blood triglycerides [29].

*NPC1* is a gene implicated in Niemann–Pick disease, an autosomal recessive lipid storage disorder characterized by progressive neurodegeneration caused by over-accumulation of cholesterol and glycosphingolipids in late endosomal/lysosomal compartments. Interestingly, when *Npc1* +/− male mice were fed a high-fat diet, they deposited more fat, were heavier, and developed adipocyte hypertrophy [30,31]. A recent study performed in humans with obesity reported that *NPC1* expression was significantly higher in the subcutaneous and omental white adipose tissue, and it was lower after weight loss [32]. The mutated amino acid that we found is totally conserved among species. Various genetic studies have implicated *NPC1* in its susceptibility to obesity, and it is associated with early-onset and morbid adult obesity [33,34,35]. This gene may be a promising candidate for a gene that modifies the lipedema phenotype or a gene important in the differential diagnosis of lipedema.

### 3.3. Mutations in Candidate Genes

The truncating variant in *INSR*, c.3079C>T; p.Arg1027* (rs121913144), is a good candidate to explain the lipedema phenotype. In fact, heterozygous mutations in *INSR* have been associated with familial hyperinsulinemic hypoglycemia [36], and a patient with recurrent hypoglycemia has been reported to have bilateral lower limb lipedema [37]. We have also found a rare missense variant predicted to be deleterious in the same gene, c.3262C>T; p.Arg1088Cys (rs867075117), in another subject.

*RYR1* encodes a ryanodine receptor found in skeletal muscle that functions as a calcium release channel in the sarcoplasmic reticulum. Deleterious missense variants in *RYR1* have been found in obese patients [38]. In pigs, a *RYR1* variant has been associated with changes in subcutaneous fat distribution [39]. Interestingly, we have found a total of five variants, three of them in a single patient, all rare and predicted to be deleterious.

Finally, we found a rare deleterious variant in *PPARA*, c.875A>G; p.Lys292Arg (rs773411072). This is a very interesting variant as it has a minor allele frequency of 0.0008%, and, in a cohort of only 162 subjects, we found two carriers. Lipedema is an inflammatory disease [15,40]. *PPARA* activation prevents inflammation in white adipose tissue [41], therefore a mutation in this gene could increase inflammation in lipedema tissue. This may indicate an involvement of *PPARA* in the development of lipedema.

## 4. Discussion

The familial nature of the condition suggests that lipedema is genetically determined. The inheritance of lipedema might be either X-linked dominant, autosomal dominant with sex limitation, and/or oligogenic [2,42].

In this work, we identified 21 heterozygous variants predicted deleterious by 3 predictors out of 5, in 17 subjects. Interestingly, most of the genes carrying deleterious variants are involved in steroidogenesis, lipid homeostasis, and the insulin signaling pathway. For example, *PLIN1* encodes the protein perilipin-1 that covers lipid storage droplets in adipocytes, protecting them until they can be broken down by hormone-sensitive lipases [26]. Another gene, *LIPE*, encodes a lipase that is expressed in steroidogenic tissues and converts cholesteryl esters to free cholesterol for steroid hormone production. One isoform is expressed in adipose tissue, where it hydrolyses stored triglycerides to free fatty acids [43]. We hypothesize that a single heterozygous variant could cause a less severe phenotype with only localized fat tissue accumulation as in lipedema versus generalized obesity. Finally, *PPARG*-encoded protein is a key regulator of adipocyte differentiation and glucose homeostasis and controls fatty acid beta-oxidation in peroxisomes. GHR antagonism or lack of GH function causes, in mice, an increase in subcutaneous adipose tissue similar to lipedema in humans [44].

We identified predicted deleterious variants in genes associated with lipodystrophy in patients with lipedema. Therefore, it might be possible that some forms of partial lipodystrophy are in fact lipedema, or some forms of lipedema could in fact be partial lipodystrophy. Therefore, our panel may be important when considering a differential or additional diagnosis for lipedema, and to expand the phenotypic spectrum of genes associated with lipodystrophy that might be able to cause lipedema.

The ketogenic diet and other low-carbohydrate diets are popular with women who have lipedema [45]. The *INSR* gene encodes the insulin receptor and plays a key role in the carbohydrate metabolism; it is also important in adipocyte differentiation where it induces the expression of *PPARG*. Insulin, after binding its receptor, stimulates glucose and fatty acid transport and lipid synthesis, and suppresses lipolysis. Antagonists of the insulin receptor have been developed to improve metabolic outcomes [46]. Changes in carbohydrate metabolism pathways may be important in the maintenance of lipedema tissue.

*NPC1* encodes a protein important for the transport of cholesterol and fatty acids from lysosomes to other cellular compartments and is responsible for maintaining intracellular cholesterol homeostasis. *NPC1* is highly expressed in human white adipose tissue adipocytes [35]. Because of the involvement of *NPC1* in obesity [35], this gene can be considered in the differential diagnosis of lipedema.

*RYR1* encodes a calcium channel that mediates the release of Ca^2+^ from the sarcoplasmic reticulum into the cytoplasm and triggers muscle contraction. It is also required for normal embryonic development of skeletal muscle, heart, skin, and bones [47]. From a porcine model, *RYR1* is also involved in fat accumulation and distribution [39].

*POMC* encodes the preproopiomelanocortin protein. It is a complex propeptide encoding a range of melanocortin peptides that are released by tissue-specific proteolytic processing. POMC-producing hypothalamic neurons regulate body weight. There is an important role of the primary cilia in the neuronal circuit development of the POMC neurons of the arcuate nucleus of the hypothalamus neurons and they may serve as a critical node that bridges poor early-life nutritional conditions to adult-life obesity and metabolic disorders [48].

*NR0B2* encodes an orphan receptor that contains a putative ligand-binding domain but lacks a DNA-binding domain. This receptor is a member of the nuclear hormone receptor family. It is able to inhibit estrogen receptor function [49]. *NR0B2* is associated with subcutaneous fat tissue accumulation in mildly obese individuals. Therefore, the analysis of this gene could be considered for differential diagnosis in people with lipedema.

*GCKR* encodes a regulatory protein that inhibits glucokinase in liver and pancreatic islet cells. In addition, it recruits GCK to the nucleus with their reciprocal affinity modulated by fructose metabolites [50]. Polymorphisms in the *GCKR* gene are associated with fatty liver [51]. Fatty liver and subsequent forms of liver disease increase the amount of thoracic duct lymph formed in patients [52]. Increasing the lymph load may increase the risk of developing lipedema, and therefore, this gene could be considered as a contributing factor to lipedema.

PPAR transcription factors belong to the steroid hormone receptor superfamily and affect the expression of target genes involved in cell proliferation, cell differentiation, and in immune and inflammation responses. *PPARA* encodes a ligand-activated transcription factor, a key-regulator of lipid metabolism that regulates the beta-oxidation of fatty acids in peroxisomes [53].

Based on our results, we recommend the sequencing of a panel of genes that have an association with isolated lipedema, syndromic subcutaneous fat tissue accumulation, or with partially overlapping diseases for differential diagnosis [13] (see Supplementary Table S1 with the subdivision of the genes in subpanels). If genes associated with isolated lipedema, syndromic subcutaneous fat tissue accumulation, or differential diagnosis do not carry variants that could explain the phenotype, candidate genes are analyzed to find novel associations or new gene diseases.

## 5. Conclusions

In conclusion, we developed a NGS panel comprising genes that may be important for the differential diagnosis of lipedema, as well as candidate genes to help identify genes involved in the etiopathogenesis of lipedema [2]. In this panel, we have included genes known to be involved in the onset of isolated subcutaneous fat accumulation, genes involved in lipodystrophy and obesity, which have some overlapping features with lipedema, and genes involved in syndromic fat accumulation in order to evaluate for concurrent conditions or a differential diagnosis for conditions that may look similar to lipedema [2,3,13]. In addition, the panel includes candidate genes because of the pathway in which they are involved and for their importance in mouse models [2,3,13].

With this work, we also show that genes involved in partial lipodystrophy or obesity may carry deleterious variants that are important in lipedema patients. Therefore, these genes may be considered in the differential diagnosis of lipedema or for broadening or narrowing the phenotypic spectrum, and highlights the molecular diagnosis in addition to the clinical diagnosis.

Finally, similarly for patients with partial lipodystrophy that can be treated with leptin-replacement therapy [54,55], and patients with growth hormone deficiency that show a reduction in subcutaneous and visceral fat mass after growth hormone replacement therapy [56], knowing the genes and pathways dysregulated in patients with lipedema is fundamental to find targets for pharmacological and other treatments.

## Figures and Tables

**Table 1:** Variants identified in the analyzed patients. All variants were heterozygous. * = Premature stop codon; B = benign; D = deleterious; DC = disease causing; LP = likely pathogenic; NR = not reported; P = pathogenic; PoD = possibly damaging; PD = probably damaging; T = tolerated; VUS = variant of unknown significance. Transcript isoforms: *PLIN1* = NM_001145311.2; *LIPE* = NM_005357.4; *ALDH18A1* = NM_002860.4; *PPARG* = NM_015869.5; *GHR* = NM_000163.5; *INSR* = NM_000208.4; *RYR1* = NM_000540.3; *NPC1* = NM_000271.5; *POMC* = NM_000939.4; *NR0B2* = NM_021969.3; *GCKR* = NM_001486.4; *PPARA* = NM_001001928.3.

	Patient	Gene	Nucleotide Change	Amino Acid Change	SNP ID	MAF (%)	MutationTaster	SIFT	Polyphen-2	CADD	VarSome	Stage/Sex
Differential diagnosis genes	1	*PLIN1*	c.722T>C	p.Leu241Pro	rs914779001	NR	DC	D	PD	24.9	VUS	3/F
	2	*LIPE*	c.1141C>T	p.Arg381Cys	rs772317492	0.0004	DC	D	PD	31	VUS	3/F
	3	*PPARG*	c.1424C>T	p.Thr475Met	rs1479145908	NR	DC	D	PD	26	LP	2/F
	4	*POMC*	c.616G>T	p.Glu206 *	rs202127120	0.05	DC	/	/	39	P	3/F
	5	*NR0B2*	c.265C>T	p.Gln89 *	rs150160927	0.004	DC	/	/	35	VUS	3/F
		*GCKR*	c.1135dup	p.Thr379Asnfs *36	rs573498430	0.1	DC	/	/	24.2	VUS	
	6	*NPC1*	c.3011C>T	p.Ser1004Leu	rs150334966	0.07	DC	T	B	23.4	LP	3/F
	7	*NPC1*	c.2903A>G	p.Thr968Met	rs773767253	0.002	DC	T	B	20.8	P	2/F
Syndromic genes	8	*ALDH18A1*	c.2276C>T	p.Thr759Ile	rs781126562	0.003	DC	D	PD	29.2	LP	3/F
	9	*ALDH18A1*	c.1233G>T	p.Leu411Phe	rs758828421	0.0008	DC	D	PoD	24.8	LP	3/F
	10	*GHR*	c.293G>A	p.Trp98*	rs1237134960	0.0008	DC	/	/	39	P	2/F
Candidate genes	11	*INSR*	c.3079C>T	p.Arg1027*	rs121913144	0.0004	DC	/	/	42	P	3/F
	12	*INSR*	c.3262C>T	p.Arg1088Cys	rs867075117	0.0008	DC	D	PD	29.5	LP	3/F
	13	*RYR1*	c.341G>A	p.Arg114His	rs574357386	0.008	Pol	D	PD	27.9	LP	3/F
	14	*RYR1*	c.947G>A	p.Arg316His	rs193922761	0.001	DC	D	PD	32	LP	NA/F
			c.10097G>A	p.Arg3366His	rs137932199	0.09	DC	T	B	24.3	LP	
			c.11798A>G	p.Tyr3933Cys	rs147136339	0.08	DC	D	PD	32	LP	
	15	*RYR1*	c.1967C>T	p.Thr656Met	rs4802472	0.0008	DC	D	PD	24.7	LP	3/F
	16	*PPARA*	c.875A>G	p.Lys292Arg	rs773411072	0.0008	DC	D	PD	28.1	VUS	1/F
	17	*PPARA*	c.875A>G	p.Lys292Arg	rs773411072	0.0008	DC	D	PD	28.1	VUS	3/F

## Data Availability

Data is contained within the article.

## Supplementary Materials

The following supporting information can be downloaded at: https://www.mdpi.com/article/10.3390/jpm12020268/s1, Table S1: Genes analyzed in the present study. The genes are subdivided in three subpanels: isolated lipedema, syndromic subcutaneous fat tissue accumulation, differential diagnosis, candidate genes. ALMS = Alstrom syndrome; BBS = Bardet-Biedl syndrome; BDVS = Blakemore-Durmaz-Vasileiou syndrome; BFLS = Borjeson-Forssman-Lehmann syndrome; CDLS = Cornelia de Lange syndrome; CGL = congenital generalized lipodystrophy; CRPT = Carpenter syndrome; FPLD = Familial partial lipodystrophy; HHF = Familial hyperinsulinemic hypoglycemia; HKLLS = Hennekam lymphangiectasia-lymphedema syndrome; LMPHM = Lymphatic malformation; LPHDST = Lymphedema-distichiasis; MODY = Maturity-onset diabetes of the young; PPNAD = primary pigmented nodular adrenocortical disease; PHP = Pseudohypoparathyroidism; WBS = Williams-Beuren syndrome.
