# Supplementary material for: A Multi-Gene Panel to Identify Lipedema-Predisposing Genetic Variants by a Next-Generation Sequencing Strategy
Source: J Pers Med. 2022 Feb 11;12(2):268. doi: 10.3390/jpm12020268 (PMC8877075; doi:10.3390/jpm12020268)
Supplement: Supplementary file 1 [file jpm-12-00268-s001.zip › jpm-1544385-supplementary.pdf]

Subpanel	Gene	OMIM gene ID	Disease/Mouse model/Cellular function	OMIM disease ID	Reference/GeneCards link/MGI ID (if not in OMIM)
Isolated lipedema	*AKR1C1*	*600449	Lipedema	/	doi:10.3390/ijms21176264
Syndromic subcutaneous fat tissue accumulation	*ALDH18A1*	*138250	Cutis laxa, fat pads and retinopathy	/	doi:10.1016/j.ejpn.2014.01.003
	NSD1	*606681	Sotos syndrome with diabetes, asthma, lipedema	/	doi:10.1016/j.ejmg.2009.06.001
	POU1F1	*173110	Combined pituitary deficiency and lipedema	/	doi:10.1055/s-0029-1224154
	TBL1XR1	*608628	Pierpont syndrome	#602342	
Differential diagnosis	*ADCY3*	*600291	Severe obesity	/	doi:10.1038/s41588-017-0023-6
	AKT2	*164731	*AKT2*-linked lipodystrophy	/	doi:10.1016/j.ecl.2017.01.012
	ADRA2A	*104210	Atypical familial partial lipodystrophy	/	doi:10.1172/jci.insight.86870
	AGPAT2	*603100	FPLD1	#608594	
	CAV1	*601047	FPLD7	#606721	
	CIDEC	*612120	FPLD5	#615238	
	LIPE	*151750	FPLD6	#615980	
	LMNA	*150330	FPLD2	#151660	
	MFN2	*608507	*MFN2*-associated lipomatosis	/	doi:10.1016/j.jacl.2018.07.009
	PALB2	*610355	Familial multiple subcutaneous lipomatosis	/	doi:10.1016/j.hemonc.2016.01.001
	PLIN1	*170290	FPLD4	#613877	
	PPARG	*601487	FPLD3<br>Obesity, severe	#604367<br>#601665	
	CCBE1	*612753	HKLLS1	#235510	
	FLT4	*136352	LMPHM1	#153100	
	FOXC2	*602402	LPHDST	#153400	
	GATA2	*137295	Primary lymphedema with myelodysplasia	#614038	
	GJA1	*121014	Oculodentodigital syndrome and primary lymphedema	/	doi:10.1111/cge.12158

	HGF	*142409	Primary lymphedema	/	doi:10.1089/lrb.2008.1524
	ALMS1	*606844	ALMS	#203800	
	ARL6	*608845	BBS3	#600151	
	BBIP1	*613605	BBS18	#615995	
	BBS1	*209901	BBS1	#209900	
	BBS10	*610148	BBS10	#615987	
	BBS12	*610683	BBS12	#615989	
	BBS2	*606151	BBS2	#615981	
	BBS4	*600374	BBS4	#615982	
	BBS5	*603650	BBS5	#615983	
	BBS7	*607590	BBS7	#615984	
	PTHB1	*607968	BBS9	#615986	
	C8orf37	*614477	BBS21	#617406	
	CEP19	*615586	Morbid obesity and spermatogenic failure	#615703	
	CEP290	*610142	BBS14	#615991	
	DYRK1B	*604556	Abdominal obesity-metabolic syndrome 3	#615812	
	GNAS	*139320	PHP1A<br>PHP1B<br>PHP1C<br>pseudoPHP	#103580<br>#603233<br>#612462<br>#612463	
	HDAC8	*300269	CDLS5	#300882	
	IFT172	*607386	BBS20	#619471	
	IFT27	*615870	BBS19	#615996	
	INPP5E	*613037	Mental retardation, truncal obesity, retinal dystrophy, micropenis	#610156	
	INSR	*147670	HHF5	#609968	
	KSR2	*610737	Obesity, insulin resistance, impaired cellular fuel oxidation	/	doi:10.1016/j.cell.2013.09.058
	LEP	*164160	Morbid obesity	#614962	
	LEPR	*601007	Morbid obesity	#614963	
	LZTFL1	*606568	BBS17	#615994	
	MC3R	*155540	Non-syndromic early-onset obesity	/	doi:10.1038/s41366-019-0357-5
	MC4R	*155541	Obesity	#618406	

	MEGF8	*604267	CRPT2	#614976	
	MKKS	*604896	BBS6	#605231	
	MKS1	*609883	BBS13	#615990	
	NR0B2	*604630	Early-onset mild obesity	#601665	
	PCSK1	*162150	Obesity with impaired prohormone processing	#600955	
	PHF6	*300414	BFLS	#301900	
	POMC	*176830	Obesity, adrenal insufficiency, red hair	#609734	
	PPP1R3A	*600917	Severe insulin resistance	#125853	
	RAB23	*606144	CRPT1	#201000	
	SDCCAG8	*613524	BBS16	#615993	
	SH2B1	*608937	Severe early-onset obesity	/	doi:10.1172/JCI62696
	SIM1	*603128	Obesity	/	doi:10.1007/s11695-019-04184-w
	TRIM32	*602290	BBS11	#615988	
	TTC8	*608132	BBS8	#615985	
	VPS13B	*607817	Cohen syndrome	#216550	
	WDPCP	*613580	BBS15	#615992	
Candidate genes	*A2M*	*103950	Inflammatory cytokines inhibitor	/	doi:10.1152/ajpregu.00335.2014
	ABCC6	*603234	Pseudoxanthoma elasticum	#264800	
	ABCG1	*603076	Cellular lipid homeostasis regulation	/	https://www.genecards.org/cgi-bin/carddisp.pl?gene=ABCG1&keywords=ABCG1
	ACBD7	/	Medium- and long-chain acyl-CoA esters binding	/	https://www.genecards.org/cgi-bin/carddisp.pl?gene=ACBD7&keywords=ACBD7
	ACSL1	*152425	Increased body fat mass	/	MGI:102797
	ACVR1C	*608981	Body fat distribution	/	doi:10.1210/clinem/dgab877
	ADIG	*611396	Abnormal adipose tissue development	/	MGI:2675492
	ADIPOQ	*605441	Adiponectin deficiency	#612556	
	ADRB2	+109690	Obesity, type 2 diabetes	/	https://www.genecards.org/cgi-bin/carddisp.pl?gene=ADRB2&keywords=ADRB2
	ADRB3	*109691	Increased total body fat amount	/	MGI:87939
	AEBP1	*602981	Abnormal fat cell morphology	/	MGI:1197012
	AGRP	*602311	Abnormal energy expenditure	/	MGI:892013
	AKR1B1	*103880	Steroid hormones metabolism	/	https://www.genecards.org/cgi-

					bin/carddisp.pl?gene=AKR1B1&keywords=AKR1B1
	AKR1B10	*604707	Detoxification of dietary and lipid-derived unsaturated carbonyls	/	https://www.genecards.org/cgi-bin/carddisp.pl?gene=AKR1B10&keywords=AKR1B10
	AKR1B15	*616336	Steroid hormones metabolism	/	https://www.genecards.org/cgi-bin/carddisp.pl?gene=AKR1B15&keywords=AKR1B15
	AKR1C2	*600450	Regulation of estrogens and androgens metabolism	/	https://www.genecards.org/cgi-bin/carddisp.pl?gene=AKR1C2&keywords=AKR1C2
	AKR1C3	*603966	Steroid hormones metabolism	/	https://www.genecards.org/cgi-bin/carddisp.pl?gene=AKR1C3&keywords=AKR1C3
	AKR1C4	*600451	Steroid hormones metabolism	/	https://www.genecards.org/cgi-bin/carddisp.pl?gene=AKR1C4&keywords=AKR1C4
	AKR1E2	*617451	Steroid hormones metabolism	/	https://www.genecards.org/cgi-bin/carddisp.pl?gene=AKR1E2&keywords=AKR1E2
	ANGPTL4	*605910	Abnormal lipid homeostasis<br>Abnormal lymphatic vessel morphology	/	MGI:1888999
	ANK2	*106410	Increased fat cell size	/	MGI:88025
	ANKRD26	*610855	Regulator of adipogenesis and feeding behavior	/	https://www.genecards.org/cgi-bin/carddisp.pl?gene=ANKRD26&keywords=ANKRD26
	ANXA1	*151690	Abnormal adipose tissue morphology and physiology	/	MGI:96819
	APOA1	*107680	Promotion of cholesterol efflux from tissues	/	https://www.genecards.org/cgi-bin/carddisp.pl?gene=APOA1&keywords=APOA1
	APOE	*107741	Lipid transport between organs via the plasma and interstitial fluids	/	https://www.genecards.org/cgi-bin/carddisp.pl?gene=APOE&keywords=APOE
	ARNTL	*602550	Dyslipidemia, ectopic fat formation, altered energy homeostasis	/	MGI:1096381
	ARRDC3	*612464	Body mass and energy expenditure regulation	/	doi:10.1016/j.cmet.2011.08.011
	ATG12	*609608	Increased body fat mass	/	MGI:1914776
	ATXN1	*601556	Association with ectopic-fat	/	doi: 10.1038/ng.3738
	BAIAP3	*604009	Behavior and food intake regulation	/	https://www.genecards.org/cgi-bin/carddisp.pl?gene=BAIAP3&keywords=BAIAP3
	BDNF	*113505	Body weight gain	/	doi:10.1007/s11064-021-03523-7

	BECN2	*615687	Increased body weight	/	MGI:2684950
	BRD2	*601540	Increased total body fat amount	/	MGI:99495
	BRS3	*300107	Regulation of metabolic rate, glucose metabolism	/	https://www.genecards.org/cgi-bin/carddisp.pl?gene=BRS3&keywords=BRS3
	BSCL2	*606158	CGL2	#269700	
	CADM2	*609938	Obesity	/	doi:10.1038/s41598-019-43861-9
	CAMKK2	*615002	Increased percent body fat/body weight	/	MGI:2444812
	CARTPT	*602606	Susceptibility to obesity	#601665	
	CAVIN1	*603198	CGL4	#613327	
	CD300E	*609801	Neutrophil-mediated inflammation in adipose tissue	/	doi:10.1371/journal.pone.0125718
	CDKN1A	*116899	Increased white fat cell number	/	MGI:104556
	CDKN1B	*600778	Increased white fat cell number	/	MGI:104565
	CIDEA	*604440	Role in thermogenesis and lipolysis	/	https://www.genecards.org/cgi-bin/carddisp.pl?gene=CIDEA&keywords=CIDEA
	CLOCK	*601851	Increased body weight	/	MGI:99698
	CNR1	*114610	Role in diet-induced obesity, dyslipidemia, liver steatosis, lipogenesis, energy expenditure, feeding behavior	/	https://www.genecards.org/cgi-bin/carddisp.pl?gene=CNR1&keywords=CNR1
	CNTNAP2	*604569	Diet-induced obesity.	/	doi:10.1007/s00335-012-9400-8
	COL3A1	*120180	Adipose tissue inflammation	/	MGI:88453
	CPE	*114855	BDV syndrome	#619326	
	CPEB4	*610607	Obesity	/	doi:10.1155/2018/3848560
	CRY2	*603732	Glucose and lipid metabolism modulation	/	https://www.genecards.org/cgi-bin/carddisp.pl?gene=CRY2&keywords=CRY2
	CYP19A1	*107910	Synthesis of cholesterol, steroids and other lipids	/	https://www.genecards.org/cgi-bin/carddisp.pl?gene=CYP19A1&keywords=CYP19A1
	DNAAF1	*613193	Morbid obesity	/	doi:10.3390/genes5030709
	EBF1	*164343	Regulation of lipid metabolism	/	https://www.genecards.org/cgi-bin/carddisp.pl?gene=EBF1&keywords=EBF1
	ELN	*130160	WBS	#194050	doi:10.1002/ajmg.a.30400
	ENPP1	*173335	Susceptibility to obesity	#601665	
	EPAS1	*603349	Insulin resistance and obesity	/	doi:10.3390/life11060552

	ESR1	*133430	Increased white fat cell number and size	/	MGI:1352467
	ESRRA	*601998	Regulation of lipid metabolism	/	https://www.genecards.org/cgi-bin/carddisp.pl?gene=ESRRA&keywords=ESRRA
	FABP2	*134640	Intracellular transport of long-chain fatty acids and their acyl-CoA esters, triglyceride-rich lipoprotein synthesis, lipid sensor for energy homeostasis	/	https://www.genecards.org/cgi-bin/carddisp.pl?gene=FABP2&keywords=FABP2
	FABP4	*600434	Lipid transport in adipocytes. Delivery of long-chain fatty acids to their receptors in the nucleus	/	https://www.genecards.org/cgi-bin/carddisp.pl?gene=FABP4&keywords=FABP4
	FFAR4	*609044	Susceptibility to obesity	#607514	
	FGF21	*609436	Glucose uptake stimulation in differentiated adipocytes	/	https://www.genecards.org/cgi-bin/carddisp.pl?gene=FGF21&keywords=FGF21
	FOXO1	/	Regulation of metabolic homeostasis	/	https://www.genecards.org/cgi-bin/carddisp.pl?gene=FOXO1&keywords=FOXO1
	FTO	*610966	Susceptibility to obesity	#612460	
	FZD9	*601766	Modulation in adipose tissue by chronic hyperadiponectinemia	/	doi:10.1371/journal.pone.0067712
	GCKR	*600842	Glucokinase regulation by forming an inactive complex with glucokinase	/	https://www.genecards.org/cgi-bin/carddisp.pl?gene=GCKR&keywords=GCKR
	GDF15	*605312	Food intake, energy expenditure, body weight regulation	/	https://www.genecards.org/cgi-bin/carddisp.pl?gene=GDF15&keywords=GDF15
	GDF3	*606522	Adipose-tissue homeostasis, energy balance regulation	/	https://www.genecards.org/cgi-bin/carddisp.pl?gene=GDF3&keywords=GDF3
	GFRAL	*617837	Food intake, energy expenditure, body weight regulation	/	https://www.genecards.org/cgi-bin/carddisp.pl?gene=GFRAL&keywords=GFRAL
	GHR	*600946	Increased total body fat amount	/	MGI:95708
	GHRL	*605353	Susceptibility to obesity	#601665	
	GHSR	*601898	Energy homeostasis and body weight regulation	/	https://www.genecards.org/cgi-bin/carddisp.pl?gene=GHSR&keywords=GHSR
	GPD1	*138420	Critical role in carbohydrate and lipid metabolism	/	https://www.genecards.org/cgi-bin/carddisp.pl?gene=GPD1&keywords=GPD1
	GPR26	*604847	Increased susceptibility to diet-induced	/	MGI:2441758

			obesity with increased food intake		
	GPR82	*300748	Abnormal adipose tissue amount	/	MGI:2441734
	GPRC6A	*613572	Coordination of nutritional and hormonal signals through anabolic steroids	/	https://www.genecards.org/cgi-bin/carddisp.pl?gene=GPRC6A&keywords=GPRC6A
	GRB14	*601524	Insulin receptor signaling inhibitor that regulates growth and metabolism	/	https://www.genecards.org/cgi-bin/carddisp.pl?gene=GRB14&keywords=GRB14
	GRIK1	*138245	Severe early-onset obesity	/	doi:10.1371/journal.pgen.1006657
	GRPR	*305670	Contribution to the regulation of food intake	/	https://www.genecards.org/cgi-bin/carddisp.pl?gene=GRPR&keywords=GRPR
	GSDMB	*611221	Adipocyte development and differentiation	/	doi:10.1038/ng.3738
	GSK3A	*606784	Negative regulator in the hormonal control of glucose homeostasis	/	https://www.genecards.org/cgi-bin/carddisp.pl?gene=GSK3A&keywords=GSK3A
	GSK3B	*605004	Negative regulator of glucose homeostasis involved in energy metabolism, inflammation, mitochondrial dysfunction	/	https://www.genecards.org/cgi-bin/carddisp.pl?gene=GSK3B&keywords=GSK3B
	GUCY2C	*601330	Increased subcutaneous adipose tissue amount	/	MGI:106903
	H6PD	*138090	Cortisone reductase deficiency 1	#604931	
	HDAC4	*605314	High *HDAC4* expression in visceral adipose tissue in obese. HDAC4 is negatively correlated with pro-inflammatory cytokines	/	doi:10.1080/13813455.2020.1734843
	HIF1A	*603348	HIF-1α activity contributes to chronic inflammation in obesity.	/	doi:10.1152/ajpendo.00626.2010
	HIPK2	*606868	Essential regulator of white fat development	/	doi:10.1073/pnas.1322275111
	HMGA2	*600698	Role in adipogenesis and mesenchymal differentiation. Knockout study in mouse shows that *HMGA2* is involved in diet-induced obesity	/	https://www.genecards.org/cgi-bin/carddisp.pl?gene=HMGA2&keywords=HMGA2
	HOXA1	*142955	Expression linked with fat accumulation in brown adipocytes	/	doi:10.1016/j.gep.2016.01.002
	HOXA10	*142957	Expression linked with fat accumulation in brown adipocytes	/	doi:10.1016/j.gep.2016.01.002

	HOXA2	*604685	Expressed in adipose tissue	/	doi:10.1210/jc.2012-2953
	HOXA4	*142953	Expression linked with fat accumulation in brown adipocytes	/	doi:10.1016/j.gep.2016.01.002
	HOXA5	*142952	Expression linked with fat accumulation in brown adipocytes	/	doi:10.1016/j.gep.2016.01.002
	HOXB4	*142965	Upregulated during adipocyte development	/	doi:10.1186/gb-2010-11-8-r80
	HOXB8	*142963	Expressed in adipose tissue	/	doi:10.1210/jc.2012-2953
	HOXC13	*142976	Body fat distribution modulation	/	doi:10.1038/ng.685
	HOXC4	*142974	Expressed in adipose tissue	/	https://www.genecards.org/cgi-bin/carddisp.pl?gene=HOXC4&keywords=HOXC4
	HOXC8	*142970	Expressed in adipose tissue	/	doi:10.1210/jc.2012-2953
	HOXD4	*142981	Adipocytes differentiation	/	doi:10.3389/fphys.2014.00479
	IFI35	*600735	Inflammation regulation	/	https://www.genecards.org/cgi-bin/carddisp.pl?gene=IFI35&keywords=IFI35
	IFT74	*608040	BBS22	#617119	
	IL18	*600953	Increased white adipose tissue amount	/	MGI:107936
	IL6	*147620	Increases lipolysis, improves insulin resistance, regulates energy and glucose homeostasis	/	https://www.genecards.org/cgi-bin/carddisp.pl?gene=IL6&keywords=IL6
	IL6R	*147880	Energy and glucose homeostasis regulation	/	https://www.genecards.org/cgi-bin/carddisp.pl?gene=IL6R&keywords=IL6R
	INSIG2	*608660	Negative regulator of cholesterol biosynthesis	/	https://www.genecards.org/cgi-bin/carddisp.pl?gene=INSIG2&keywords=INSIG2
	IRS1	+147545	Susceptibility to type 2 diabetes mellitus	#125853	
	IRX3	*612985	Energy metabolism regulator	/	https://www.genecards.org/cgi-bin/carddisp.pl?gene=IRX3&keywords=IRX3
	ITGA9	*603963	Abnormal lymph circulation	/	MGI:104756
	KDM3A	*611512	Involved in obesity resistance through regulation of metabolic genes	/	https://www.genecards.org/cgi-bin/carddisp.pl?gene=KDM3A&keywords=KDM3A
	KLF16	*606139	Lipid metabolism regulation	/	doi:10.1136/gutjnl-2020-321774
	KRAS	*190070	Lymphedema in developmental syndromes	/	doi:10.1038/ejhg.2015.175

	LPIN1	*605518	Required for adipocyte differentiation	/	https://www.genecards.org/cgi-bin/carddisp.pl?gene=LPIN1&keywords=LPIN1
	LRP2	*600073	Critical component of the hypothalamic feeding regulatory pathway	/	doi:10.1038/ncomms2896
	LRRC8A	*608360	Abnormal fat cell morphology	/	MGI:2652847
	LYPLAL1	*616548	Obesity	/	doi:10.1016/j.mce.2015.05.001.
	MAPK11	*602898	Inflammatory response	/	https://www.genecards.org/cgi-bin/carddisp.pl?gene=MAPK11&keywords=MAPK11
	MAPK12	*602399	Inflammatory response	/	https://www.genecards.org/cgi-bin/carddisp.pl?gene=MAPK12&keywords=MAPK12
	MAPK13	*602899	Inflammatory response	/	https://www.genecards.org/cgi-bin/carddisp.pl?gene=MAPK13&keywords=MAPK13
	MAPK14	*600289	Inflammatory response	/	https://www.genecards.org/cgi-bin/carddisp.pl?gene=MAPK14&keywords=MAPK14
	MC1R	*155555	Negative regulation of inflammatory response	/	https://www.genecards.org/cgi-bin/carddisp.pl?gene=MC1R&keywords=MC1R
	MCHR1	*601751	Feeding behaviors and energy metabolism control	/	https://www.genecards.org/cgi-bin/carddisp.pl?gene=MCHR1&keywords=MChR1
	MED13	*603808	Increased susceptibility to obesity and worsened glucose intolerance when fed a high fat diet in mice	/	MGI:3029632
	MEIS1	*601739	Inactivation required for adipogenesis	/	doi:10.1016/j.celrep.2018.09.086.
	MLXIPL	*605678	Abnormal glucose and lipid homeostasis	/	MGI:1927999
	MMP19	*601807	Increased fat cell size	/	MGI:1927899
	MRAP2	*615410	Susceptibility to obesity	#615457	
	MSX2	*123101	Adipogenic differentiation suppressor	/	doi:10.1074/jbc.M306972200
	NBEA	*604889	Abnormal interscapular fat pad morphology	/	MGI:1347075
	NCOA1	*602691	Required for energy control balance between white and brown adipose tissues and for mediating steroid hormone response	/	https://www.genecards.org/cgi-bin/carddisp.pl?gene=NCOA1&keywords=NCOA1
	NCOA2	*601993	Required for energy control balance	/	https://www.genecards.org/cgi-

			between white and brown adipose tissues and for mediating steroid hormone response		bin/carddisp.pl?gene=NCOA2&keywords=NCOA2
	NCOA3	*601937	Involved in the coactivation of nuclear receptors (steroids, retinoids, prostanoids	/	https://www.genecards.org/cgi-bin/carddisp.pl?gene=NCOA3&keywords=NCOA3
	NDN	*602117	Prader-Willi syndrome	#176270	
	NEGR1	*613173	Predisposition to obesity	#601665	
	NEIL1	*608844	Homozygous null mice develop severe obesity, dyslipidemia, fatty liver disease, hyperinsulinemia	/	MGI:1920024
	NF1	*613113	Neurofibromatosis type 1 with lymphedema	/	doi:10.3892/ol.2016.4469
	NGEF	*605991	Association with abdominal visceral fat	/	doi:10.1371/journal.pone.0137564
	NMU	*605103	Pain, stress, inflammation and feeding regulation	/	https://www.genecards.org/cgi-bin/carddisp.pl?gene=NMU&keywords=NMU
	NPC1	*607623	Obesity	/	doi:10.2337/db16-0877
	NPY	*162640	Control of feeding and secretion of gonadotrophin-release hormone	/	https://www.genecards.org/cgi-bin/carddisp.pl?gene=NPY&keywords=NPY
	NPY4R	*601790	Obesity	/	doi:10.1002/oby.21435
	NR1D1	*602408	Regulation of lipid and bile acid metabolism, adipogenesis, gluconeogenesis, macrophage inflammatory response	/	https://www.genecards.org/cgi-bin/carddisp.pl?gene=NR1D1&keywords=NR1D1
	NR2F2	*107773	Abnormal glucose homeostasis and lipid level	/	MGI:1352452
	NR2F6	*132880	Adipocyte differentiation inhibition	/	doi:10.1074/mcp.M114.045328
	NR3C1	*138040	Negative regulation of adipogenesis	/	https://www.genecards.org/cgi-bin/carddisp.pl?gene=NR3C1&keywords=NR3C1
	NTRK2	*600456	Obesity, hyperphagia, and developmental delay	#613886	
	OGG1	*601982	Maintenance of metabolic homeostasis	/	doi:10.1016/j.dnarep.2019.102667
	OMA1	*617081	Lipid metabolism regulation	/	https://www.genecards.org/cgi-bin/carddisp.pl?gene=OMA1&keywords=OMA1

	OSBPL8	*606736	Lipid transporter involved in lipid countertransport between the endoplasmic reticulum and the plasma membrane	/	https://www.genecards.org/cgi-bin/carddisp.pl?gene=OSBPL8&keywords=OSBPL8
	PANX1	*608420	Adipose stromal cell differentiation and fat accumulation	/	doi:10.1038/s41598-018-34234-9.
	PAX6	*607108	Pancreatic islet alpha cells differentiation. Glucagon, insulin and somatostatin promoters binding	/	https://www.genecards.org/cgi-bin/carddisp.pl?gene PAX6&keywords=PAX6
	PBX1	*176310	Overweight/obesity and metabolic alterations	/	doi:10.4162/nrp.2008.2.4.289.
	PDE11A	*604961	PPNAD2	#610475	
	PDE3B	*602047	Abnormalities in glycerol and fatty acid levels, changes in adipocyte morphology, decreased body fat percentage.	/	MGI:1333863
	PDX1	*600733	MODY4	#606392	
	PER1	*602260	Abnormal basal metabolism, body weight, estrous cycle, food intake, food preference	/	MGI:1098283
	PIGC	*601730	Glycosylphosphatidylinositol lipid anchor biosynthesis	/	https://www.genecards.org/cgi-bin/carddisp.pl?genePIGC&keywords=PIGC
	PIK3CA	*171834	Lymphatic malformation	/	doi:10.1371/journal.pone.0200343
	PITPNM1	*608794	Mice homozygous for a knock-out allele exhibit decrease in circulating cholesterol	/	MGI:1197524
	PNPLA2	*609059	Neutral lipid storage disease with myopathy	#610717	
	PPARGC1A	*604517	Blood pressure, cellular cholesterol homoeostasis control, development of obesity	/	https://www.genecards.org/cgi-bin/carddisp.pl?genePPARGC1A&keywords=PPARGC1A
	PPARGC1B	*608886	Increased risk of obesity.	/	https://www.genecards.org/cgi-bin/carddisp.pl?genePPARGC1B&keywords=PPARGC1B
	PRDM16	*605557	Differentiation of brown adipose tissue	/	https://www.genecards.org/cgi-bin/carddisp.pl?genePRDM16&keywords=PRDM16
	PRDM2	*601196	Specific effector of estrogen action	/	https://www.genecards.org/cgi-bin/carddisp.pl?genePRDM2&keywords=PRDM2
	PRKAA1	*602739	Cellular energy sensor	/	https://www.genecards.org/cgi-

					bin/carddisp.pl?genePRKAA1&keywords=PRKAA1
	PRKAA2	*600497	Cellular energy sensor	/	https://www.genecards.org/cgi-bin/carddisp.pl?genePRKAA2&keywords=PRKAA2
	PRKAB1	*602740	Cellular energy sensor	/	https://www.genecards.org/cgi-bin/carddisp.pl?genePRKAB1&keywords=PRKAB1
	PRKAB2	*602741	Cellular energy sensor	/	https://www.genecards.org/cgi-bin/carddisp.pl?genePRKAB2&keywords=PRKAB2
	PRKACA	*601639	Cellular energy sensor	/	https://www.genecards.org/cgi-bin/carddisp.pl?genePRKACA&keywords=PRKACA
	PRKACB	*176892	Cellular energy sensor	/	https://www.genecards.org/cgi-bin/carddisp.pl?genePRKACB&keywords=PRKACB
	PRKAG1	*602742	Cellular energy sensor	/	https://www.genecards.org/cgi-bin/carddisp.pl?genePRKAG1&keywords=PRKAG1
	PRKAG2	*602743	Cellular energy sensor	/	https://www.genecards.org/cgi-bin/carddisp.pl?genePRKAG2&keywords=PRKAG2
	PRKAG3	*604976	Skeletal muscle glycogen content and metabolism QTL	#619030	https://www.genecards.org/cgi-bin/carddisp.pl?genePRKAG3&keywords=PRKAG3
	PRKAR1A	*188830	Carney complex, type 1	#160980	
	PRKAR2A	*176910	Regulation of lipid and glucose metabolism	/	https://www.genecards.org/cgi-bin/carddisp.pl?genePRKAR2A&keywords=PRKAR2A
	PRKAR2B	*176912	Regulation of lipid and glucose metabolism	/	https://www.genecards.org/cgi-bin/carddisp.pl?genePRKAR2B&keywords=PRKAR2B
	PROX1	*601546	Lymphedema	/	doi:10.1002/mgg3.1424
	PRRC2A	*142580	Insulin-dependent diabetes mellitus	/	https://www.genecards.org/cgi-bin/carddisp.pl?genePRRC2A&keywords=PRRC2A
	PTN	*162095	Proliferation promotion of adipocytes precursor cells	/	doi:10.1126/scisignal.aag0487
	PYY	*600781	Pancreatic secretion, gut mobility and energy homeostasis regulation	/	https://www.genecards.org/cgi-bin/carddisp.pl?genePYY&keywords=PYY
	RARB	*180220	Association with carbohydrate intake	/	doi:10.1038/s41380-018-0079-4
	RB1	*614041	Retinoblastoma and lipomatosis	/	doi:10.5999/aps.2014.41.6.785.
	RBP4	*180250	Adipokine	/	doi:10.1530/EJE-11-0431.
	RETN	*605565	Susceptibility to noninsulin-dependent	#125853	

			diabetes mellitus		
	RGS2	*600861	Brown adipose tissue function and differentiation balance	/	doi:10.1016/j.molmet.2019.09.015
	RREB1	*602209	Brown adipocyte differentiation	/	https://www.genecards.org/cgi-bin/carddisp.pl?geneRREB1&keywords=RREB1
	RSC1A1	*601966	Homozygous null mice develop obesity, increased serum cholesterol and leptin levels and increased absorption of D-glucose in the small intestine.	/	MGI:3526447
	RSPO3	*610574	Impact on body fat distribution, adipose cell biology regulation	/	doi:10.1038/s41467-020-16592-z
	RYR1	*180901	Hypothalamic expression	/	doi:10.1152/physiolgenomics.00006.2015
	SCD	*604031	Lipid biosynthesis, mitochondrial fatty acid oxidation regulation, body energy homeostasis	/	https://www.genecards.org/cgi-bin/carddisp.pl?geneSCD&keywords=SCD
	SCPEP1	/	Association with intramuscular fat in pigs	/	doi:10.1111/jbg.12189
	SDC3	*186357	Association with obesity	#601665	
	SERPINA6	*122500	Major transport protein for glucocorticoids and progestins in the blood	/	https://www.genecards.org/cgi-bin/carddisp.pl?geneSERPINA6&keywords=SERPINA6
	SFRP1	*604156	Increased adiposity, dysregulated glucose metabolism, enhanced macrophage infiltration in deficient mice	/	doi:10.1371/journal.pone.0078320
	SFRP5	*604158	Anti-inflammatory adipokine involved in obesity, type 2 diabetes mellitus	/	doi:10.1111/jcmm.15023
	SIRT1	*604479	Sensor of the cytosolic ratio of NAD(+)/NADH, influenced by glucose deprivation and caloric restriction	/	https://www.genecards.org/cgi-bin/carddisp.pl?geneSIRT1&keywords=SIRT1
	SIRT6	*606211	Glucose homeostasis regulation	/	https://www.genecards.org/cgi-bin/carddisp.pl?geneSIRT6&keywords=SIRT6
	SKP2	*601436	Adipocyte proliferation control	/	doi:10.1074/jbc.M608144200
	SLC13A5	*608305	Utilization of circulating citrate facilitation for the generation of metabolic energy and the synthesis of fatty acids and cholesterol	/	https://www.genecards.org/cgi-bin/carddisp.pl?geneSLC13A5&keywords=SLC13A5
	SLC2A4	*138190	Insulin-regulated facilitative glucose	/	https://www.genecards.org/cgi-

			transporter		bin/carddisp.pl?geneSLC2A4&keywords=SLC2A4
	SLC35D3	*612519	Mutation in this gene causes metabolic syndrome	/	doi:10.1371/journal.pgen.1004124
	SLCO4C1	*609013	Severe early-onset obesity	/	doi:10.1371/journal.pgen.1006657
	SNAP25	*600322	Metabolic disease	/	doi:10.1016/j.neuroscience.2018.07.035
	SNRPN	*182279	Prader-Willi syndrome	#176270	
	SREBF1	*184756	Cholesterol biosynthesis and lipid homeostasis regulation	/	https://www.genecards.org/cgi-bin/carddisp.pl?geneSREBF1&keywords=SREBF1
	STAB1	*608560	Low density lipoprotein endocytosis regulation	/	https://www.genecards.org/cgi-bin/carddisp.pl?geneSTAB1&keywords=STAB1
	STAT3	*102582	Melanocortin production and body energy homeostasis regulation	/	https://www.genecards.org/cgi-bin/carddisp.pl?geneSTAT3&keywords=STAT3
	STAT5A	*601511	Adipogenesis promotion	/	doi:10.1007/s12199-010-0193-7
	STAT5B	*604260	Impaired glucose tolerance, increased circulating triglyceride level, increased total body fat amount	/	MGI:103035
	STRA6	*610745	In obesity, downregulation in adipocytes and adipose stromovascular fraction	/	doi:10.1128/MCB.01106-13
	SYPL2	/	Susceptibility to morbid obesity	/	doi:10.1038/ejhg.2014.255
	TAF7L	*300314	Abnormal brown adipose tissue morphology, thermogenesis, differentiation	/	MGI:1921719
	TBC1D1	*609850	Involved in the insulin-stimulated glucose uptake into cells	/	https://www.genecards.org/cgi-bin/carddisp.pl?geneTBC1D1&keywords=TBC1D1
	TBC1D4	*612465	Noninsulin-dependent diabetes mellitus 5	#616087	
	TBX15	*604127	Expression positively correlated with glycolytic metabolism, inversely correlated with obesity in subcutaneous and visceral white adipose tissue	/	doi:10.2337/db17-0218
	TGFB1	*190180	Browning of white fat	/	doi:10.1016/j.molmet.2018.07.008
	TGM2	*190196	A homozygous null mutation causes alterations in glucose and aerobic energy metabolism	/	MGI:98731
	TMEM18	*613220	KO homozygous mice exhibit increase in	/	MGI:2387176

			body weight, fat mass, susceptibility to diet-induced obesity, increased food intake		
	TNXB	*600985	Involved in fat deposition in sheep	/	doi:10.1186/s12864-018-4747-1
	TRH	*613879	Increased circulating cholesterol, creatinine, HDL cholesterol levels	/	MGI:98823
	TRIM72	*613288	Mediation of the degradation of the insulin receptor and insulin receptor substrate 1. When upregulated, metabolic syndrome featuring insulin resistance, obesity, hypertension, dyslipidaemia	/	doi:10.1038/nature11834
	TRPV1	*602076	Increased body weight	/	MGI:1341787
	TTR	*176300	Transthyretin antisense oligonucleotides improve insulin sensitivity in obese mice	/	doi:10.2337/db14-0970
	TUB	*601197	Retinal dystrophy and obesity	#616188	
	TYK2	*176941	Abnormal body temperature homeostasis, brown adipose tissue morphology, fat cell differentiation, mitochondrial physiology, decreased energy expenditure, impaired adaptive thermogenesis, glucose tolerance, increased body weight, cholesterol level, circulating free fatty acids level, circulating insulin level, obesity	/	MGI:1929470
	UBE2E2	*602163	Associated with ectopic-fat traits	/	doi:10.1038/ng.3738
	UCP1	*113730	Thermogenic respiration	/	https://www.genecards.org/cgi-bin/carddisp.pl?geneUCP1&keywords=UCP1
	UCP2	*601693	Susceptibility to obesity	#607447	
	UCP3	*602044	Thermogenesis and energy balance	/	https://www.genecards.org/cgi-bin/carddisp.pl?geneUCP3&keywords=UCP3
	VEGFA	*192240	Key factor in the regulation of angiogenesis in adipose tissue	/	doi:10.1038/s41598-017-16686-7
	VEGFC	*601528	Lymphatic malformation 4	#615907	
	WDR13	*300512	KO mice increase pancreatic islet mass and higher serum insulin levels, and are mildly obese	/	https://www.genecards.org/cgi-bin/carddisp.pl?geneWDR13&keywords=WDR13

	WNT10B	*601906	Molecular switch for adipogenesis	/	https://www.genecards.org/cgi-bin/carddisp.pl?geneWNT10B&keywords=WNT10B
	WNT11	*603699	Obesity-induced adipose tissue inflammation and metabolic dysfunction promotion	/	doi:10.2337/db14-1164
	WNT4	*603490	Adipocyte differentiation promotion	/	doi:10.1016/j.febslet.2008.08.011
	YWHAZ	*601288	Insulin sensitivity regulation	/	https://www.genecards.org/cgi-bin/carddisp.pl?geneYWHAZ&keywords=YWHAZ
	ZEB1	*189909	Impaired glucose tolerance, increased body weight, circulating adiponectin level, circulating corticosterone level, circulating free fatty acids level, circulating insulin level, circulating leptin level, circulating triglyceride level, percent body fat/body weight, increased total body fat amount, insulin resistance	/	MGI:1344313
	ZMPSTE24	*606480	Mandibuloacral dysplasia with type B lipodystrophy	#608612	
	ZNF423	*604557	Adipogenic commitment control in mice, dysregulationed in human hypertrophic obesity	/	doi:10.1007/s00125-017-4471-4
	ZNRF3	*612062	Inhibition of Wnt/β-catenin signaling,	/	doi:10.3389/fphys.2018.00792

Supplementary Table S1. Genes analyzed in the present study. The genes are subdivided in three subpanels: isolated lipedema, syndromic subcutaneous fat tissue accumulation, differential diagnosis, candidate genes. ALMS = Alstrom syndrome; BBS = Bardet-Biedl syndrome; BDVS = Blakemore-Durmaz-Vasileiou syndrome; BFLS = Borjeson-Forssman-Lehmann syndrome; CDLS = Cornelia de Lange syndrome; CGL = congenital generalized lipodystrophy; CRPT = Carpenter syndrome; FPLD = Familial partial lipodystrophy; HHF = Familial hyperinsulinemic hypoglycemia; HKLLS = Hennekam lymphangiectasia-lymphedema syndrome; LMPHM = Lymphatic malformation; LPHDST = Lymphedema-distichiasis; MODY = Maturity-onset diabetes of the young; PPNAD = primary pigmented nodular adrenocortical disease; PHP = Pseudohypoparathyroidism; WBS = Williams-Beuren syndrome.
